# Aerobic capacity and mitochondrial function in bipolar disorder: a longitudinal study during acute phases and after clinical remission

**DOI:** 10.3389/fpsyt.2024.1386286

**Published:** 2024-03-26

**Authors:** Anna Giménez-Palomo, Mariona Guitart-Mampel, Gemma Roqué, Ester Sánchez, Roger Borràs, Ana Meseguer, Francesc Josep García-García, Esther Tobías, Laura Valls-Roca, Gerard Anmella, Marc Valentí, Luis Olivier, Oscar de Juan, Iñaki Ochandiano, Helena Andreu, Joaquim Radua, Norma Verdolini, Michael Berk, Eduard Vieta, Glòria Garrabou, Josep Roca, Xavier Alsina-Restoy, Isabella Pacchiarotti

**Affiliations:** ^1^ Department of Psychiatry and Psychology, Institute of Neuroscience, Hospital Clínic de Barcelona, Barcelona, Catalonia, Spain; ^2^ Bipolar and Depressive Disorders Unit, Institut d’Investigacions Biomédiques August Pi I Sunyer (IDIBAPS), Barcelona, Catalonia, Spain; ^3^ Biomedical Research Networking Centre Consortium on Mental Health (CIBERSAM), Instituto de Salud Carlos III, Madrid, Spain; ^4^ Department of Medicine, School of Medicine and Health Sciences, Institute of Neurosciences (UBNeuro), University of Barcelona (UB), Barcelona, Catalonia, Spain; ^5^ Inherited Metabolic Diseases and Muscular Disorders Research Lab, Cellex-IDIBAPS, Faculty of Medicine and Health Sciences-University of Barcelona, Internal Medicine Department-Hospital Clinic of Barcelona and Centro de Investigación en Red de Enfermedades Raras (CIBERER), Catalonia, Spain; ^6^ Pneumology Department (ICR), Hospital Clínic de Barcelona, Institut d'Investigacions Biomèdiques August Pi i Sunyer (IDIBAPS), School of Medicine, University of Barcelona, Centro de Investigación en Red de Enfermedades Respiratorias (CIBERES), Barcelona, Catalonia, Spain; ^7^ Cardiovascular Institute, Hospital Clínic, Institut d'Investigacions Biomèdiques August Pi i Sunyer (IDIBAPS), Universitat Autònoma de Barcelona, Barcelona, Spain; ^8^ Local Health Unit Umbria 1, Department of Mental Health, Mental Health Center of Perugia, Perugia, Italy; ^9^ IMPACT, The Institute for Mental and Physical Health and Clinical Translation, School of Medicine, Deakin University, Geelong, VIC, Australia; ^10^ Centre for Youth Mental Health, Florey Institute for Neuroscience and Mental Health and the Department of Psychiatry, Orygen, The University of Melbourne, Melbourne, Australia

**Keywords:** bipolar disorder, mania, depression, aerobic capacity, endurance time, mitochondrial respiration

## Abstract

**Background:**

Aerobic capacity has shown to predict physical and mental health-related quality of life in bipolar disorder (BD). However, the correlation between exercise respiratory capacity and mitochondrial function remains understudied. We aimed to assess longitudinally intra-individual differences in these factors during mood episodes and remission in BD.

**Methods:**

This study included eight BD patients admitted to an acute psychiatric unit. Incremental cardiopulmonary exercise test (CPET) was conducted during acute episodes (T0), followed by constant work rate cycle ergometry (CWRCE) to evaluate endurance time, oxygen uptake at peak exercise (VO_2peak_) and at the anaerobic threshold. The second test was repeated during remission (T1). Mitochondrial respiration rates were assessed at T0 and T1 in peripheral blood mononuclear cells.

**Results:**

Endurance time, VO_2peak_, and anaerobic threshold oxygen consumption showed no significant variations between T0 and T1. Basal oxygen consumption at T1 tended to inversely correlate with maximal mitochondrial respiratory capacity (r=-0.690, p=0.058), and VO_2peak_ during exercise at T1 inversely correlated with basal and minimum mitochondrial respiration (r=-0.810, p=0.015; r=-0.786, p=0.021, respectively).

**Conclusions:**

Our preliminary data showed that lower basal oxygen consumption may be linked to greater mitochondrial respiratory capacity, and maximum oxygen uptake during the exercise task was associated with lower basal mitochondrial respiration, suggesting that lower oxygen requirements could be associated with greater mitochondrial capacity. These findings should be replicated in larger samples stratified for manic and depressive states.

## Introduction

Bipolar disorder (BD) is a chronic and recurrent disease characterized by depressive and manic or hypomanic mood episodes alternated with periods of euthymia (1). It is associated with reduced functionality and medical comorbidities (1), especially metabolic disorders that impact physical and mental health prognosis (2). Individuals with BD face an elevated risk of premature cardiovascular-related death, attributed in part to a reduced exercise capacity (3).

Exercise tolerance has been widely studied in somatic diseases. Endurance time during constant work rate cycle ergometry (CWRCE), known as the total time the individual maintains exercise at a constant work rate, has been associated with patients’ experience of physical functioning in daily life, and considered a useful efficacy endpoint in clinical intervention trials (4). Poor aerobic endurance and muscle strength are associated in mental illnesses, including BD, with impaired physical function, increased risk of lifestyle-related diseases, and early mortality (5). In BD, longer illness duration, higher body mass index (BMI), higher levels of depression and lower physical activity levels have been associated with lower physical fitness, emerging as an eminent modifiable risk factor for somatic comorbidity (3).

Cardiorespiratory fitness, assessed through maximum oxygen uptake (VO_2peak_), is considered a predictive measure for cardiovascular disease and premature mortality, demonstrating the potential of exercise to counteract compromised physical health in BD (3).

Exercise-induced changes in mitochondrial function, crucial for determining exercise capacity (6), have been explored in various contexts but remain understudied in BD, despite suggestions of mitochondrial dysfunction as a potential marker in the disorder’s pathophysiology (7, 8).

No biomarkers have yet been implemented in clinical practice to support diagnostic and therapeutic processes. This study addresses a gap in research by longitudinally assessing intra-individual differences in aerobic capacity and mitochondrial respiration during different mood states in BD patients. We primarily hypothesized a positive correlation between aerobic capacity and mitochondrial respiration in BD, and secondarily an improvement in both after clinical remission compared to the acute episode. Therefore, we aimed to study the association between respiratory capacity during physical activity and mitochondrial respiration, measured after the extraction of peripheral blood mononuclear cells (PBMC), across the different states of the illness, and also longitudinally determine differences in the aerobic capacity and oxygen uptake between the acute mood episodes and remission. Finally, we studied the association between aerobic capacity with BMI and physical activity.

## Methods

### Study design and population

The current work, derived from a financed longitudinal study (PI21/00169), aimed to assess intra-individual differences in oxygen consumption, exercise capacity and mitochondrial function longitudinally in patients with BD (9).

Adult inpatients admitted to our acute psychiatric unit with BD type I with an acute manic or depressive episode, according to DSM-5 criteria (10), were eligible for this study. Assessments occurred during the acute episode (T0) and after symptomatic remission (T1), defined as standardized clinical scores ≤7 at the Young Mania Rating Scale (YMRS) (11) or the 17-item Hamilton Depression Rating Scale (HDRS) (12) (i.e. symptoms absent or nearly absent), before hospital discharge. A disease course shorter than ten years was necessary for patient recruitment. Patients with intellectual quotient lower than 80, with substance use disorders other than tobacco and cannabis, and with any cardiac, respiratory, auto-immune, inflammatory illness or with an acute infectious illness were excluded from this study, as well as those with known history of familial mitochondrial disease.

The capacity to provide informed consent was assessed before entering the study and re-assessed after remission. This study was approved by the Hospital Clínic Research Ethics Committee (HCB/2021/0358).

### Clinical evaluation

Socio-demographic and clinical data were collected. Lung function was assessed with forced spirometry and diffusing lung capacity for carbon monoxide (DLCO), to exclude any respiratory limitations (13). Manic and depressive symptoms were assessed respectively using standardized psychometric scales: YMRS (11) and HDRS (12). Functioning was assessed with Functioning Assessment Short Test (FAST) (14), disease severity with Clinical Global Impression Scale – Severity (CGI-S) (15), and physical activity with International Physical Activity Questionnaire (IPAQ) (16), and adherence to Mediterranean diet with a 17-score scale (PREDIMED-17) (17). At T1, CGI-Improvement (CGI-I) scale was obtained, and HDRS, YMRS, FAST, IPAQ, CGI-S were re-administered. Clinical variables were assessed at T0 and T1.

### Assessment of aerobic capacity

At T0, patients were evaluated with an incremental CPET, and then with a CWRCE (18), both conducted on a cycle ergometer (Lode Corival CEPT mod:960900, Groningen, The Netherlands). During the CWRCE, patients performed the test at the 80% of the peak work-load achieved in the incremental CPET. The CWRCE was also conducted at T1.

In the CWRCE, endurance time in seconds, an indicator of aerobic capacity (6), VO_2peak_ and oxygen uptake at the anaerobic threshold in L/min were measured. The last was obtained as an expression of the exercise intensity indicating the transition from mild to moderate exercise and from aerobic to anaerobic work intensity. The association between the aerobic capacity with BMI and physical activity was studied to assess the influence of non-psychiatric factors, such as physical fitness and exercise routines, in exercise performance.

### Assessment of mitochondrial respiration

Mitochondrial oxygen consumption rates (OCRs) were assessed at T0 and T1, after the isolation of peripheral blood mononuclear cells (PBMC) obtained by a Ficoll density gradient centrifugation procedure. To determine OCRs at T0 and T1, a million living PBMCs resuspended in PBS1x were used. High-resolution respirometry was conducted in fresh cells at 37°C by polarographic oxygen sensors in a two-chamber Oxygraph-2k system (OROBOROS Instruments, Innsbruck, Austria). Specific OCRs were obtained (Routine: basal oxygen consumption with no exogenous substrates; Leak: oxygen consumption not coupled to ATP synthesis; ETC: maximal capacity of the electron transport chain; and Rox: oxygen consumption not linked to mitochondrial activity). Routine, Leak and ETC OCRs were registered by subtracting the rates from Rox as it is considered unspecific and non-mitochondrial oxygen consumption. The following inhibitors and uncouplers were manually injected: (i) oligomycin (1.5 mM), an ATP synthase inhibitor, (ii) carbonyl cyanide 3-chlorophenylhydrazone (CCCP) (1 mM), a mitochondrial uncoupler, and (iii-iv) rotenone (2 mM) and antimycin A (0.2 mM), which are complex I and complex III inhibitors, respectively.

Oxygen uptake was normalized per million cells. Results are expressed as picomoles of oxygen per million cells (pmol O_2_/million).

### Statistical analysis

Statistical analyses were computed with ‘IBM SPSS Statistics 25’ and GraphPad Prism. Quantitative variables were summarized as median and interquartile range (IQR), and categorical variables as frequencies. For intra-subjects’ comparisons, Wilcoxon matched-pairs signed rank tests were used. Spearman test was used for correlation analyses. Results were controlled for pharmacological treatment. Statistical significance level was set at p<0.05.

## Results

**Table 1** outlines the key characteristics of the 8 participants (6 manic, 2 depressed) included.

**Table 1:** Socio-demographic and clinical characteristics of the sample.

	Mania	Depression
	n = 6 (75%)	n = 2 (25%)
Sex, n females (%)	4 (66.7)	1 (50.0)
Age, mean (SD)	24.2 (4.4)	33.5 (9.2)
BMI (T0), mean (SD)	21.0 (1.2)	22.7 (1.9)
Abdominal circumference (T0), mean cm (SD)	76.7 (5.3)	92.0 (8.5)
Number of total previous episodes, mean (SD)	1.8 (1.2)	3.5 (2.1)
Months from onset of first affective episode, mean (SD)*	48.3 (36.5)	192.0 (67.9)
Age of onset of BD, mean (SD)	22.2 (5.2)	30.5 (13.4)
Age at first hospitalization, mean (SD)	22.0 (4.5)	28.0 (17.0)
Number of previous psychiatric hospitalizations, mean (SD)	1.0 (1.3)	2.5 (3.5)
Number of previous manic episodes, mean (SD)	0.5 (1.2)	1.0 (1.4)
Number of previous depressive episodes, mean (SD)	0.8 (1.0)	2.5 (0.7)
Previous psychiatric diagnosis, n (%)	4 (66.7)	2 (100)
Previous psychiatric medication, n (%)	3 (50.0)	2 (100)
Current psychiatric follow-up, n (%)	3 (50.0)	2 (100)
Life stressor, n (%)	2 (33.3)	0 (0.0)
Psychotic symptoms, n (%)	5 (83.3)	0 (0.0)
Delusions, n (%)	4 (66.7)	0 (0.0)
Hallucinations, n (%)	2 (33.3)	0 (0.0)
Previous suicide attempt, n (%)	1 (16.7)	1 (50.0)
Cannabis use, n (%)	4 (66.7)	0 (0.0)
Somatic illness, n (%)	1 (16.7)	0 (0.0)
Family psychiatric history, n (%)	4 (66.7)	2 (100)
Family history of BD, n (%)	1 (16.7)	1 (50.0)
Treatment discontinuation, n (%)	2 (33.3)	0 (0.0)
Living alone, n (%)	1 (16.7)	1 (50.0)
Higher education, n (%)	1 (16.7)	2 (100)
Working activity, n (%)	3 (50.0)	2 (100)

SD, standard deviation; BD, bipolar disorder. *Significant at p<0.05.

Compared to patients with a manic episode, those with a depressive episode presented higher HDRS total score at admission (25.0 ± 11.3 vs 4.0 ± 2.7), with no major differences at endpoint. As expected, those with a manic episode displayed higher YMRS total score at admission compared to depressive patients (23.5 ± 10.5 vs 1.5 ± 2.1), which partially reduced at discharge (4.8 ± 2.0 vs 0.0 ± 0.0). In addition, relevant differences were found between groups in FAST total score at admission (13.3 ± 8.5 in mania vs 37.5 ± 3.5 in depression) and after clinical remission (6.0 ± 7.1 in mania vs 29.5 ± 4.9 in depression), with no major differences in CGI, IPAQ or PREDIMED-17 scales scores.

Spirometry was performed in all patients to assess resting pulmonary capacity, which was within reference values in all cases.

**Figure 1:**
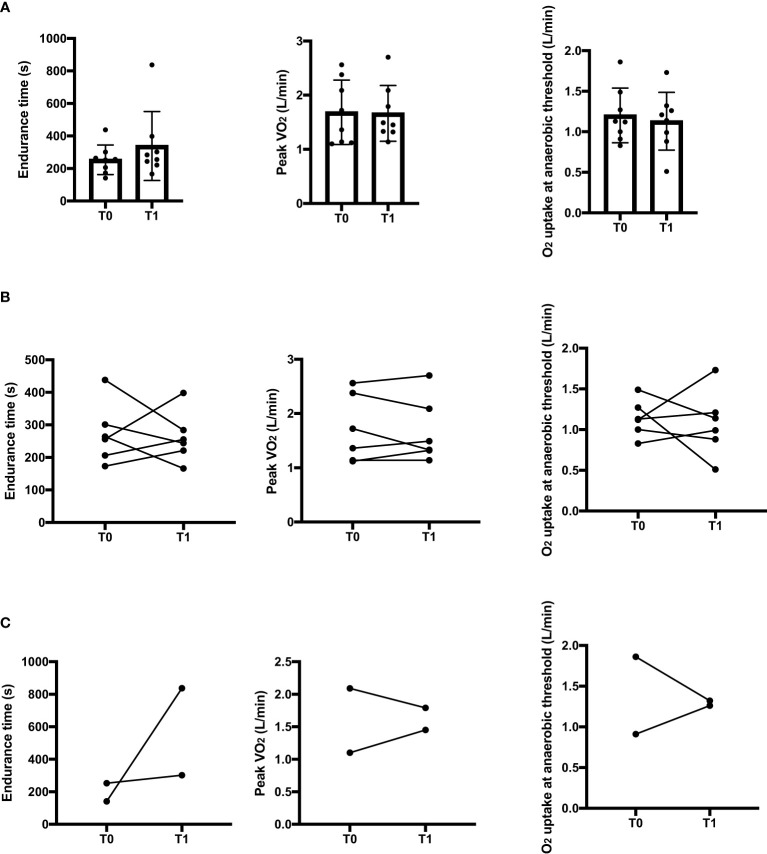
Intra-subject comparisons in endurance time, peak oxygen uptake and oxygen uptake at the anaerobic threshold between the acute state (T0) and clinical remission (T1) for the overall sample **(A)**, for manic patients **(B)** and for depressive patients **(C)** in the constant work rate cycle ergometry. s, seconds; L, liters.

Intra-subject longitudinal comparisons between acute mood episodes (T0) and clinical remission (T1) for the overall patients’ sample are shown in **Figure 1**.

No significant differences between T0 and T1 were found in the endurance (p=0.779), VO_2peak_, (p=0.779) or oxygen consumption at the anaerobic threshold (p=0.726).

All patients included had normal weight (median: 21.20 kg/m^2^, IQR: 20.39-22.61). However, patients’ BMI tended to be directly correlated with endurance time at T1 (r=0.667, p=0.071). IPAQ total score at T0 was associated with more prolonged endurance time (r=0.905, p=0.005).

Despite mitochondrial respiratory capacity showed a tendency to increase at T1 compared to T0, differences in the different OCRs were not significant.

Endurance time at T0 or T1 did not show any association with mitochondrial respiratory capacity. Likewise, at T0, no relevant correlations were observed between oxygen uptake during the effort test and mitochondrial oxygen consumption. Nevertheless, at T1, basal oxygen consumption (before starting CWRCE) tended to be inversely correlated with maximum mitochondrial respiratory capacity (ETC) (r=-0.690, p=0.058). In addition, VO_2peak_ at T1 was inversely correlated with Routine (r=-0.810, p=0.015) and Leak (r=-0.786, p=0.021) OCRs.

## Discussion

In this preliminary study, no significant differences were found in aerobic capacity, including endurance time, VO_2peak_ or oxygen consumption at the anaerobic threshold during CWRCE, or mitochondrial respiration between severe acute mood states and after clinical remission, although the second showed a tendency to increase in clinical remission compared to the acute states, which is supported by recent results from our group (9). An inverse association was noted between basal oxygen consumption and maximum mitochondrial respiratory capacity after remission, suggesting individuals with increased mitochondrial capacity might have lower basal oxygen requirements and higher mitochondrial efficiency, which should be confirmed in larger samples.

In addition, the maximum oxygen uptake during CWRCE at clinical remission was inversely correlated with basal mitochondrial respiration, suggesting physical fitter individuals may exhibit lower resting mitochondrial oxygen requirements. Our results suggest an association between an electron transport chain dysfunction and an impaired aerobic respiration, which could be a risk factor for an increased anaerobic respiration and oxidative stress.

Higher BMI tended to be correlated with longer endurance time after clinical remission, hinting a better physical fitness in this cohort. Also, IPAQ total score was associated with more prolonged endurance time during the acute state, revealing a stronger association of endurance capacity with physical fitness rather than with the severity of the acute episode.

To the authors’ knowledge, this is the first study reporting intra-subject longitudinal differences in BD between acute states and clinical remission and aiming to find a potential association between oxygen consumption capacity during an effort test and mitochondrial respiration. Other studies assessing mitochondrial OCRs in mood disorders have been conducted with smaller samples (19, 20).

The study strengths include intra-individual comparisons between clinical states in patients with short course of illness and severe episodes, larger samples compared to previous studies, and *in vivo* mitochondrial respiratory capacity assessment. Also, laboratory measurements were performed at the same time as the clinical evaluation and the effort tests in the cycle ergometer were obtained. Limitations include the small sample size, explained by the severity of mood episodes, which hindered recruitment. The small sample size did not allow differentiation from participants at index mania and index depression, which might be expected to differ. Finally, even though the inpatient unit ensures a lower variability in different environmental conditions, since illicit substances and tobacco are strictly forbidden, and a balanced diet is provided in all cases, other factors, such as some individual characteristics, might have influenced mitochondrial respiration.

In conclusion, our results suggest that impaired mitochondrial oxygen consumption capacity may be reflected by exercise performance, and that physical fitness might predict a better exercise performance over the illness’ state, whereas mitochondrial respiratory capacity might increase in clinical remission compared to the acute states. Further studies should elucidate aerobic exercise could enhance mitochondrial respiratory capacity, whether this could be used as a state-dependent marker in the assessment of clinical response, and if the enhancement of physical activity might be a potential strategy to prevent not only metabolic comorbidities, but also mitochondrial dysfunction, which might pave the way for personalized interventions in BD.

## Data availability statement

The raw data supporting the conclusions of this article will be made available by the authors, without undue reservation.

## Ethics statement

The studies involving humans were approved by Hospital Clinic Research Ethics Committee (HCB/2021/0358). The studies were conducted in accordance with the local legislation and institutional requirements. The participants provided their written informed consent to participate in this study.

## Author contributions

AG: Conceptualization, Data curation, Formal analysis, Investigation, Methodology, Project administration, Resources, Writing – original draft, Writing – review & editing. MG: Conceptualization, Data curation, Formal analysis, Investigation, Methodology, Supervision, Visualization, Writing – original draft, Writing – review & editing. GR: Investigation, Methodology, Project administration, Resources, Supervision, Writing – review & editing. ES: Investigation, Supervision, Visualization, Writing – review & editing. RB: Data curation, Formal analysis, Investigation, Methodology, Writing – review & editing. AM: Investigation, Methodology, Project administration, Resources, Writing – review & editing. FG: Conceptualization, Data curation, Investigation, Resources, Visualization, Writing – review & editing. ET: Investigation, Methodology, Resources, Supervision, Writing – review & editing. LV: Resources, Supervision, Visualization, Writing – review & editing. GA: Conceptualization, Methodology, Software, Supervision, Writing – review & editing. MV: Conceptualization, Methodology, Visualization, Writing – review & editing. LO: Project administration, Visualization, Writing – review & editing. Od: Project administration, Visualization, Writing – review & editing. IO: Project administration, Visualization, Writing – review & editing. HA: Project administration, Visualization, Writing – review & editing. JR: Conceptualization, Investigation, Methodology, Software, Supervision, Visualization, Writing – original draft, Writing – review & editing. NV: Conceptualization, Supervision, Visualization, Writing – review & editing. MB: Conceptualization, Data curation, Investigation, Supervision, Visualization, Writing – original draft, Writing – review & editing. EV: Conceptualization, Supervision, Visualization, Writing – review & editing. GG: Writing – review & editing, Writing – original draft. JR: Writing – original draft, Writing – review & editing, Conceptualization, Data curation, Investigation, Methodology, Software, Supervision, Validation, Visualization. XA: Conceptualization, Investigation, Methodology, Software, Supervision, Validation, Visualization, Writing – review & editing, Project administration, Resources. IP: Conceptualization, Investigation, Methodology, Project administration, Resources, Supervision, Visualization, Writing – review & editing, Funding acquisition, Writing – original draft.
